# Relatively young T1D adults using fixed doses of insulin have higher diabetes distress levels in a sample of patients from a Brazilian tertiary hospital

**DOI:** 10.1186/s13098-019-0501-3

**Published:** 2019-12-12

**Authors:** M. S. V. M. Silveira, T. G. Bovi, E. J. Pavin

**Affiliations:** 1 Internal Medicine Postgraduate Program, Faculty of Medical Sciences-Unicamp, Campinas, Brazil; 2 Endocrinology Division, Department of Internal Medicine, Faculty of Medical Sciences-Unicamp, Campinas, Brazil

**Keywords:** Diabetes mellitus, Type 1, Depressive symptoms, Anxiety, Emotional distress

## Abstract

**Background:**

Elevated rates of anxiety and depressive symptoms in Type 1 Diabetes patients (T1D) and high rates of diabetes-specific distress (DD) have been shown. Several factors may be responsible for increase the DD levels such as age, life changes, lack of familiar support, education, insulin regimens (IRs) and chronic complications. The goals of this study were: 1—to compare DD levels, anxiety and depressive symptoms according to age (< and ≥ 25 years old), 2—to evaluate the association between DD levels, anxiety and depressive symptoms and IRs, and 3—to evaluate the association between DD levels, anxiety and depressive symptoms and chronic complications.

**Methods:**

In a cross-sectional study, T1D patients receiving outpatient care at Unicamp tertiary hospital were included. Inclusion criteria were age at least 18 years old and diagnosis of T1D for 6 months. Exclusion criteria were cognitive impairment, major psychiatric disorders, severe diabetes-related complications, and pregnancy. Depressive symptoms were evaluated by the depression subscale of the Hospital Anxiety and Depression Scale (HAD-D) and the anxiety symptoms by the anxiety subscale of the same instrument (HAD-A). DDS scale assessed DD. Glycemic control was evaluated by HbA1C. The latest lipid panel results were recorded and IRs and chronic complications were obtained through chart review.

**Results:**

Of all 70 patients, 70% were younger than 25 years old. No differences were found between two groups according to gender, education, and income (p = 0.39, p = 0.87, and p = 0.52, respectively). HbA1c mean was 10% in both groups (p = 0.15). Older patients had higher levels of total DD and physician DD than younger (p = 0.0048 and p = 0.0413; respectively).Total DD and DD on subscales 1 and 2 were higher in patients using fixed doses of insulin compared to variable doses according to carbohydrates count (p = 0.0392, p = 0.0383 and p = 0.0043, respectively). No differences were found between anxiety and depressive symptoms and age and IRs. Similarly, no differences were found among DD levels, anxiety and depressive symptoms in patients with and without chronic complications.

**Conclusions:**

When providing education and care for T1D patients, health providers should consider age, patient’s developmental stage, with its related demands and the burden of insulin regimen.

## Background

The emotional side of Type 1 Diabetes (T1D) is a concerned area for clinicians and researchers. Many studies have shown elevated rates of anxiety and depressive symptoms in T1D patients, as well as the presence of elevated rates of diabetes-specific distress [1–5]. The term diabetes distress (DD) defines the distress and common feelings involved in the daily life of people with diabetes [6, 7].

Trief et al. in a large heterogeneous T1D sample, showed that patients with high depressive symptoms had worse clinical outcomes compared to those without this condition [4]. In addition, Fisher et al. demonstrated that moderate and high DD levels have been associated with high HbA1c [8, 9]. These conditions may hamper the patients’ ability to manage the disease and decrease their quality of life.

The complexity of T1D treatment is responsible at least in part for many sources of the DD related to the management of the disease. Patients with clinical meaningful DD and high depressive symptoms are less likely to have the behaviors needed to manage T1D appropriately [4, 8, 9]. Moreover, other several factors may be responsible for difficulties in T1D self-management such as age range, life changes, lack of familiar support, education, ethnicity, and financial problems [10–12].

A recent systematic review showed that specifically diabetes-tailored psychological interventions are effective in reducing elevated DD and HbA1c [13] and Fisher et al. described that best results are achieved when DD is target directly for intervention [14].

The sources of depressive symptoms and emotional distress in T1D patients vary at different ages because they face distinct challenges across the adult life span [10, 11, 15]. The patient’s developmental stage, with its related demands such as work and family, psychological adjustments and the potential burden of T1D treatment and comorbidities should be evaluated carefully.

Recent research conducted in a Brazilian tertiary public hospital showed that low-income T1D patients have high rates of depressive symptoms and DD [5]. These patients face additional challenges because they don’t have access to modern insulins and tools to appropriately manage their diabetes and, furthermore, there is a lack of structured diabetes education and psychological support for T1D patients throughout the country.

To develop personalized psychological support for T1D patients at Unicamp Diabetes Clinic accordingly, we propose to investigate the emotional burden of T1D in different age groups, patients who received different insulin regimens (IRs) and patients with and without chronic complications.

The goals of this study were: 1—to compare DD levels, anxiety and depressive symptoms according to age groups (< and ≥ 25 years old), 2—to evaluate the associations between DD, anxiety and depressive symptoms levels and IRs and 3—to evaluate the associations between DD levels, anxiety and depressive symptoms and diabetes chronic complications in a T1D population followed at Unicamp tertiary hospital.

## Methods

In a cross-sectional study, patients with T1D receiving outpatient care at the Type 1 Diabetes Clinic of the University of Campinas tertiary hospital were included. Patients were interviewed between January 2016 and January 2017.

Inclusion criteria were age 18 and older and diagnosis of T1D for at least 6 months. Exclusion criteria were cognitive impairment that could affect the patients’ ability to answer the protocol questions, history of major psychiatric disorders (such as schizophrenia, drug addiction, dementia), patients with severe diabetes-related complications (blindness, on hemodialysis, limb amputations, and stroke), and pregnancy.

Patients were invited to take part in the study during routine consultations. Patients who consented to participate in this study gave permission for their clinical, laboratory and demographic data to be recorded. This study followed the principles of the Declaration of Helsinki and was approved by the University Ethics in Research Committee in December 2015 (CAAE Number: 50864815.4.0000.5404).

The evaluation of depressive symptoms was made with the depression subscale of the Hospital Anxiety and Depression Scale (HAD-D) and the anxiety symptoms were evaluated by the anxiety subscale of the same instrument (HAD-A). This scale was developed by Zigmond et al. [16] and translated and validated into Portuguese by Botega et al. [17]. Both HAD subscales have 7 items and each one is scored from 0 to 3. Bjelland et al. through a systematic literature review, identified a cut-off point of 8 for clinical relevant depression and anxiety symptoms [18].

The Diabetes Distress Scale (DDS) was used to assess DD. The DDS was developed by Polonsky et al. [7] and yields a total distress score and four subscales reflecting different sources of distress: emotional burden, physician-related distress, treatment-related distress, and interpersonal distress. The DDS has 17 items and utilizes a 6 point-Likert scale, in which the respondent indicates the presence of a problem for them, ranging from “not a problem” to a “serious problem”. Mean item scores of ≥ 2 are considered clinically meaningful [9]. This scale was validated in Brazil for the Portuguese language by Lima et al. [19]. All patients were interviewed by the same researcher, the senior author of this study, in a face to face interview. The diagnosis of T1D was clinic, in the presence of typical clinical presentation of T1D, including variable degrees of hyperglycemia, weight loss, polyuria, polydipsia, polyphagia and the need for continuous insulin use since the diagnosis.

The following variables were assessed using a questionnaire during a clinical visit: current age, age at diagnosis, T1D duration, types of prescribed insulin regimens (IRs), years of study attendance (scholarity), conjugal status, glucose self-monitoring and economic status classified by the number of Brazilian minimum wages per month, based on Brazilian Institute of Geography and Statistics classification [20]. T1D patients were divided into two income ranges: income reaching until 3 minimum wages and income above 3 minimum wages.

Glycemic level was evaluated by HbA1C, which was calculated by high-performance liquid chromatography (HPLC). We considered the last HbA1c value measured during the last clinical appointment in order to better reflect the glycemic control on the period in which the patients answered the questionnaires. The latest lipid panel results were recorded. Total cholesterol, HDL, LDL, VLDL, and triglycerides were measured by enzymatic techniques. The IRs were obtained from medical records and it was divided into three groups according to insulin protocol used: IR 1—patients using fixed doses of insulin (basal/bolus), IR 2—patients using fixed doses of insulin (basal/bolus) and were oriented to make pre-prandial correction of glycemia, IR 3—patients using variable doses of insulin (basal/bolus), according to carbohydrates counting plus pre-prandial correction of glycemia.

Chronic microvascular complications of diabetes were obtained from medical records. Diabetic retinopathy was diagnosed based on fundoscopy examinations performed by the University Ophthalmology Department. Nephropathy was diagnosed if two or more urine samples separated by at least 30 days showed “albumine/creatinine ratio above 30 mg/g”. Neuropathy was diagnosed based on annual clinical examinations performed by the staff physicians at the diabetes clinic [21].

Patients were stratified according to age groups, IRs, and the presence or absence of chronic complications. The study variables were analyzed and compared in all groups.

### Statistical methods

Descriptive analyses were done with measures of means and medians for numerical variables and frequency (percentage) for categorical variables.

Differences between groups were assessed by the Mann–Whitney test for numerical variables and by the Chi Square test or by Fisher’s exact test for categorical variables, as appropriate. Anova (mixed models) was used to compare the scales between the groups. The variables were analysed separately and with their interactions as well. Data were transformed in ranks. All analyses were undertaken using SAS version 9.4 for Windows. Statistical significance was set at 0.05.

## Results

Of all 70 patients, 70% were younger than 25 years old. Mean age in younger patients was 21.12 ± 1.65 years old and mean age in older patients was 35.42 ± 7.02 (p < 0.0001). No gender differences were found between two groups (female: 52.3% vs 63.27%; male: 47.6% vs 36.7%; p = 0.39). Also, no differences were found between two groups according to years of scholarity and income (p = 0.87 and p = 0.52, respectively). Fifty-five percent of the older patients had a partner vs 14.29% of the younger ones (p = 0.0016). HbA1c mean was 10% ± 2% in both groups (p = 0.15). The number of T1D chronic complications was higher in older patients compared to younger ones (1.00 vs 0.00; p = 0.0338). The demographic and clinical characteristics of the two groups are summarized in Table 1.

**Table 1:** Sociodemographic, clinical and laboratorial characteristics of T1D patients divided by age (Qui-square, Fisher and Mann–Whitney test)

Variable	Age < 25 (*n* = 21) Mean ± SD/median	Age ≥ 25 (*n* = 49) Mean ± SD/median	p-value
Age	21.14 ± 1.65/21.00	35.41 ± 7.02/34.00	*< 0.0001*
Scholarity	11.43 ± 3.03/12.00	11.59 ± 3.92/12.00	0.8748
Years of T1D	10.19 ± 6.26/11.00	18.55 ± 7.94/20.00	*< 0.0001*
Chronic complications	0.76 ± 0.94/0.00	1.39 ± 1.15/1.00	*0.0338*
Self-monitoring	3.00 ± 2.39/3.00	3.16 ± 2.82/3.00	0.9585
Cholesterol	167.14 ± 38.30/169.00	185.22 ± 47.08/179.00	0.2333
HDL-c	53.52 ± 10.65/53.00	52.92 ± 13.03/51.00	0.6124
LDL-c	91.10 ± 28.00/85.00	109.19 ± 45.67/98.50	0.1002
VLDL-c	111.52 ± 80.85/87.00	19.95 ± 10.65/17.00	0.8513
Triglycerides	111.52 ± 80.85/87.00	102.69 ± 67.60/86.00	0.6340
HbA1c	10% ± 2%/11%	10% ± 2%/9%	0.1547
Income	85.71%^a^; 14.29%^b^	77.55%^a^; 22.45%^b^	0.5292

Income: ^a^ income reaching until 3 Brazilian minimum wages ^b^ income above 3 Brazilian minimum wages. Values expressed as mean, SD (standard deviation), median and percentage

T1D: Type1 dabetes; age (years), time of T1D (years); scholarity (years of study); chronic complications (0–3); self-reported number of self-monitoring (measures/day); cholesterol: total cholesterol (mg/dl); HDL-c: HDL-cholesterol (mg/dl); LD-c: LDL-cholesterol (mg/dl); VLDL-c: VLDL-cholesterol (mg/dl); triglycerides (mg/dl); HbA1c: glycated hemoglobin (%)

### DD levels, anxiety and depressive symptoms according to age

Patients with age older than 25 years old had higher levels of total DD compared with patients with age < 25 years old (42.8 vs 30.4; p = 0.0048). Likewise, the older patients had higher levels of DD on subscale 2 (distress with physician) (6.94 vs 4.86; p = 0.0413).

No differences were found in anxiety symptoms rates between patients younger and older than 25 years old (8.04 ± 5.28 vs 7.33 ± 4.23; p = 0.76).

Similarly, no differences were found in depressive symptoms levels between younger and older patients (7.16 ± 5.45 vs 5.76 ± 4.04; p = 0.35). No differences were found when the interactions of factors such as age, IRs and chronic complications were analysed, according to Anova mixed models. The scores of DD, anxiety and depressive symptoms according to age are summarized in Table 2.

**Table 2:** Comparisons among DD levels, anxiety and depressive symptoms according to age (age < 25 years: n = 21; age ≥ 25 years: n = 49)—Anova

Variable	Mean ± SD/median	p-value
DDS-total		
< 25	30.48 ± 15.05/25.00	*0.0048*
≥ 25	42.84 ± 21.07/36.00	
DDS-S1		
< 25	12.14 ± 8.46/10.00	0.1821
≥ 25	14.51 ± 8.50/12.00	
DDS-S2		
< 25	4.86 ± 1.71/4.00	*0.0413*
≥ 25	6.94 ± 4.81/5.00	
DDS-S3		
< 25	12.57 ± 7.33/10.00	0.1144
≥ 25	15.41 ± 7.90/15.00	
DDS-S4		
< 25	4.95 ± 3.25/4.00	0.2048
≥ 25	6.57 ± 4.38/5.00	
HAD-A		
< 25	7.33 ± 4.23/7.00	0.7611
≥ 25	8.04 ± 5.28/7.00	
HAD-D		
< 25	5.76 ± 4.04/5.00	0.3522
≥ 25	7.16 ± 5.45/6.00	

DDS total score (DDS: Diabetes Distress Scale); DDS-S1 score (S1: emotional burden); DDS-S2 score (S2: physician distress); DDS-S3 score (S3: regimen distress); DDS-S4 score (S4: interpersonal distress); HAD-A score: anxiety symptoms (Anxiety subscale of Hospital Anxiety and Depression scale); HAD-D score: depressive symptoms (depression subscale of Hospital Anxiety and Depression Scale). Values expressed as mean, SD (standard deviation), and median

### DD levels, anxiety and depressive symptoms according to IRS (insulin regimens)

DD total score was higher in patients using IR 1 vs IR 3 (p = 0.0392). Similarly, patients using IR 1 had higher DD scores in subscale 1 compared to those using IR 3 (p = 0.0383**)**. DD score on subscale 3 was lower among patients using IR3 compared to both groups using IR 1 and IR 2 (p = 0.0043). No differences were found when the interactions of factors such as age, IRs and chronic complications were analysed, according to Anova mixed models. The scores of DD, anxiety and depressive symptoms according to IRs are summarized in Table 3.

**Table 3:** Comparisons among DD levels, anxiety and depressive symptoms according to regimen of insulin (IR 1: n = 26, IR 2: n = 29, IR 3: n = 15)—Anova

Variable	Mean ± SD/median	p-value
DDS		
IR 1	46.31 ± 23.69/43.00	0.0392*
IR 2	38.45 ± 18.01/33.00	
IR 3	28.00 ± 11.21/25.00	
DDS-S1		
IR 1	16.58 ± 8.74/15.00	0.0383*
IR 2	13.52 ± 8.62/10.00	
IR 3	9.53 ± 6.08/8.00	
DDS-S2		
IR 1	6.92 ± 5.02/4.50	0.4587
IR 2	6.10 ± 3.78/4.00	
IR 3	5.67 ± 3.66/4.00	
DDS-S3		
IR 1	18.15 ± 8.07/17.50	0.0043**
IR 2	13.79 ± 7.08/15.00	
IR 3	9.80 ± 5.73/8.00	
DDS-S4		
IR 1	7.31 ± 4.97/5.50	0.4053
IR 2	5.24 ± 3.58/3.00	
IR 3	5.69 ± 3.07/4.00	
HAD-A		
IR 1	8.23 ± 5.84/6.00	0.5052
IR 2	7.48 ± 4.70/7.00	
IR 3	7.80 ± 4.02/7.00	
HAD-D		
IR 1	7.23 ± 5.29/6.00	0.7756
IR 2	6.90 ± 5.21/7.00	
IR 3	5.60 ± 4.58/5.00	

IR 1—patients using fixed doses of insulin (basal/bolus), IR 2—patients using fixed doses of insulin (basal/bolus) plus pre-prandial correction of glycemia, IR 3—patients using variable doses of insulin (basal/bolus), according to carbohydrates counting plus pre-prandial correction of glycemia. * IR 1 DD > IR 3 DD; ** IR 1 DD and IR 2 DD > IR 3DD. DDS: Diabetes Distress Scale-total score; DDS-S1: emotional burden; DDS-S2: physician distress; DDS-S3: regimen distress; DDS-S4: interpersonal distress; HAD-A: anxiety symptoms (anxiety subscale of Hospital Anxiety and Depression Scale); HAD-D: depressive symptoms (depression subscale of Hospital Anxiety and Depression Scale). Values expressed as mean, SD (standard deviation), and median

### DD levels, anxiety and depressive symptoms according to chronic complications

No significant differences were found among DD levels in total DDS and in each subscale (total DDS: p = 0.59; S1: p = 0.70; S2: p = 0.62; S3: p = 0.83; S4: p = 0.46). Likewise, no significant differences were found among anxiety and depressive scores and chronic complications of T1D (p = 0.35 and 0.19, respectively). No differences were found when the interactions of factors such as age, IRs and chronic complications were analysed, according to Anova mixed models. The scores of DD, anxiety and depressive symptoms according to chronic complications are summarized in Table 4.

**Table 4:** Comparisons among DD levels, anxiety and depressive symptoms according to chronic complications (yes: n = 45; no: n = 25)—Anova

Variable	Complications	Mean ± SD/median	p-value
DDS			
	Yes	41.93 ± 20.29/38.00	0.5959
	No	34.08 ± 19.34/29.00	
DDS-S1			
	Yes	14.58 ± 8.21/12.00	0.7011
	No	12.40 ± 8.99/9.00	
DDS-S2			
	Yes	6.87 ± 4.95/4.00	0.6223
	No	5.32 ± 2.23/4.00	
DDS-S3			
	Yes	15.36 ± 7.63/15.00	0.8381
	No	13.12 ± 8.03/10.00	
DDS-S4			
	Yes	6.22 ± 3.73/5.00	0.4695
	No	5.84 ± 4.81/3.00	
HAD-A			
	Yes	8.00 ± 4.84/7.00	0.3559
	No	7.52 ± 5.28/7.00	
HAD-D			
	Yes	7.38 ± 5.07/7.00	0.1989
	No	5.60 ± 4.98/4.00	

DDS total score (DDS: Diabetes Distress Scale); DDS-S1 score (S1: emotional burden); DDS-S2 score (S2: physician distress); DDS-S3 score (S3: regimen distress); DDS-S4 score (S4: interpersonal distress); HAD-A score: anxiety symptoms (HAD-A: anxiety subscale of Hospital Anxiety and Depression scale); HAD-D score: depressive symptoms (HAD-D: depression subscale of Hospital Anxiety and Depression Scale). T1D chronic complications 0–3: retinopathy, nephropathy, neuropathy. Values expressed as mean, SD (standard deviation) and median

## Discussion

Given the importance of the emotional side of T1D for clinical outcomes and well being of the patients, this current study evaluated the DD levels according to age and the results demonstrated that levels of DD are higher in patients above 25 years old. As the years of T1D and number of complications was higher in older patients, the analyses were adjusted and these variables didn’t explain our findings. It is possible that life stressors such as time consumed with work and family and the lack of familial support represent a competing obstacle to conciliate the tasks of T1D management. The older patients enrolled in this study had partners more frequently than younger ones (p = 0.001) and the lifestyle of the two groups probably are different.

Developmental theorists have defined the age between 18 and 25 years old as emerging adulthood [10, 11]. In some studies, especially in developed countries, the levels of emotional distress were higher at this patient’s developmental stage [11, 15]. This was in part explained because patients in that age range face new challenges such as carrier decisions, moving out from parents’ houses to complete education and consequently the loss of parents’ supervision to manage T1D. However, this is not the reality of the majority of the patients enrolled in our current study. The majority of T1D persons followed at Unicamp tertiary hospital have low income and don’t have a higher education degree (total years of education is similar in both groups, high school degree).

Our study also showed that DD levels on DDS-subscale 2 (distress with the physician) is higher in older patients. Probably the fears of judgment by the diabetes providers due to high glucose numbers added up by the patients’ feelings of failing with T1D management are barriers in the relationship between patients and physicians, creating a negative vicious circle over time. Possibly, the older patients experienced this kind of troubling relationship for a longer time, increasing their DD levels. An important common aspect is that T1D patients frequently and repeatedly don’t bring glucometers and logbooks records to the clinical consultations. This is frustrating for physicians because it prevents insulin adjustments. Gomes et al. [22] demonstrated that the majority of T1D followed in a tertiary center in Rio de Janeiro did not have an agreement between the glycemia obtained from a glucometer and the patient’s logbook records. In addition, the main reason for this discrepancy is diet adherence, although lack of supply for strips should also be considered [22]. These patients are followed in public hospitals and receive the strips for free from the government. However, the number of strips available is insufficient to perform all the self monitorization needed. Thus, this aspect of T1D care should be carefully evaluated. Non-judgmental communication is highly recommended and relationships based on patients empowerment should be encouraged.

The current study also showed that no significant differences were found in levels of depressive and anxiety symptoms according to age groups. Emerging adults (18–25 years old) and relatively young adults (> 25 years) in the T1D population studied had similar levels of these symptoms. Moreover, as the first part of our study demonstrated that the population enrolled in the study had high rates of clinical depression and high depression symptoms [5], these conditions should be investigated in these patients, allowing adequate mental health referral and specialized treatment.

Regarding IRs, our study demonstrated that total DD was higher in patients using IR 1 (fixed doses of basal/bolus insulin). Similarly, patients using this IR had higher DD scores in subscale1 and subscale 3 (emotional burden and regimen distress, respectively). The patients using IR 3 (variable doses of insulin, according to carbohydrates counting plus pre-prandial correction of glycemia) had the lowest level of DD on subscale 3. The presence of a higher number of hypoglycemias and hyperglycemias and the need for more restrictive diet in patients belonging to IRs 1 and 2 groups, possibly account for the higher levels of DD in these patient groups. Furthermore, the use of needles in Brazil is 8 times higher when compared to the rest of the world [23], which makes it harder to adjust doses of insulin. The feelings of impotence, frustration, anger, sadness, and insecurity are expected to occur in patients with problematic glycemic control [1, 6, 9]. The presence of many episodes of hypoglycemia and the difficulty in maintaining stable glycemic control increase the suffering of T1D patients. Glycemic fluctuations affect all aspects of life [1, 4, 5, 24–27].

No significant differences were found among anxiety and depressive symptoms and IRs and chronic complications of T1D in the current study. Therefore, DD and especially the emotional burden of diabetes and the distress related to T1D management should be prioritized at Unicamp patients’ care and education.

The need for screening depression, depressive and anxiety symptoms and DD in T1D patients is well recognized. The American Diabetes Association recommends the use of validated instruments to access DD and depressive symptoms in people with diabetes and suggests that these conditions be routinely investigated [28].

Fisher et al. [14] proposed some practical strategies to guide diabetes providers in their clinical settings, addressing DD as the focus of these strategies. DD should be considered as part of having diabetes and not as comorbidity, being distinct from clinical depression. With this in mind, Fisher proposes that DD is better addressed in diabetes care and should be a part of the clinical encounters.

The interventions in DD could be simple and the use of validated instruments provides an objective way to start the conversations with T1D patients [13, 14].

The Diabetes Distress Scale (DDS) is a valid and reliable tool to access DD in patients with T1D and type 2 diabetes (T2D) [7, 14].

The higher DD scores measured in the four subscales of DDS can reveal the more distressful areas of DD which may be prioritized in psychological interventions. A specific measure of DD captures the perspective of the person with diabetes, which could facilitate communication with the patient and set personalized goals for treatment [13, 14].

The 5 questions on DDS subscale1 [7, 14]: (Feeling that diabetes is taking up too much of my mental and physical energy every day; feeling angry, scared and/or depressed when I think about living with diabetes; feeling that I will end up with serious long-term complications, no matter what I do; feeling that diabetes controls my life; feeling overwhelmed by the demands of living with diabetes) and the 5 questions on DDS subscale 3: (Not feeling confident in my day-to-day ability to manage diabetes; feeling that I am not testing my blood sugars frequently enough; feeling that I am often failing with my diabetes routine; feeling that I am not sticking closely enough to a good meal plan; not feeling motivated to keep up my diabetes self management) measure relevant feelings and behaviours related to the daily life with diabetes and its management and could be explored in the clinical conversations [14].

When necessary, patients could be referred to another and more appropriated setting if a deeply and specialized psychological approach is required.

Various types of psychological interventions have been evaluated. A recent systematic review demonstrated that diabetes-tailored psychological interventions are effective to reduce DD and HbA1c [13].

Unfortunately, the integration of effective approaches to mitigate DD and depressive symptoms in the real world of clinical care is still difficult.

We hope that the knowledge obtained through this study could contribute with the implementation of the emotional side of T1D into the strands of care and education of people with diabetes at Unicamp as well in others diabetes clinics that share the same population characteristics.

This study has some limitations. The first one is the sample size. As the study was conducted in a unique center of diabetes it was no possible to enroll a higher number of participants. Other studies with a large sample are needed. Due to a lower prevalence of T1D compared to type 2 diabetes, studies in multiple diabetes treatment centers are preferable to allow a high number of participants. Another limitation is related to the age of the participants. The older patient in this study was 47 years old. Therefore, it was no possible to determinate the levels of DD, anxiety and depressive symptoms in older adults.

The study design is another limitation because of the impossibility of evaluating DD levels, depressive and anxiety symptoms over time.

The evaluation of DD levels, anxiety and depressive symptoms and their associations with several factors affecting the daily life of T1D patients in a homogeneous sample was a strong point in this study. This issue is relevant to allow more personalized and tailored-diabetes psychological interventions.

## Conclusions

T1D relatively young adults had higher rates of total DD and physician DD (high scores on subscale 2) compared to emerging adults. The levels of anxiety and depressive symptoms were similar in both groups. Patients who received fixed doses of insulin (IR 1) had the highest levels of DD on subscales 1 and 3 (emotional burden and regimen distress).

DD, depressive symptoms and anxiety symptoms were similar in patients with and without chronic complications in the population studied.

Relatively young patients who received IR 1 should be prioritized in psychological interventions at Unicamp tertiary hospital. The questions on subscales 1, 2 and 3 could serve as a guide to start conversations with this group of patients in clinical encounters.

When providing education and care for T1D patients, health providers should consider the patient’s developmental stage, with its related demands, psychological adjustments and the burden of insulin regimen.

## Data Availability

The corresponding author can be contacted for any information related to this study.

## Abbreviations

T1D: type 1 diabetes
DD: diabetes distress
IR: insulin regimen
HAD-D: depression subscale of the Hospital Anxiety and Depression Scale
HAD-A: anxiety subscale of the Hospital Anxiety and Depression Scale
DDS: Diabetes Distress Scale
